# Madness or What?

**Published:** 1894-04-21

**Authors:** 


### The Psychologist in History.

### MADNESS OR—WHAT?—I.

### Jeanne d'Arc.

It is needless to go deeply into the historical facts of Jeanne d'Arc's life; they are known to all the world. But an inquiring and sceptical generation would fain know what was the nature of the power by which she defeated the English and reconquered France for the king. Was it, indeed, the inspiration of God, or will earthly circumstances explain her prowess in some degree?

In the first place, Jeanne d'Arc was born and brought up almost at the seat of war. The people of Domremy, her home, were on the side of the French; those of Morny, the neighbouring village, espoused the cause of the Burgundians, who were the allies of the English. The boys of the two villages indulged in fights, not altogether sham, for the honour of the parties they respectively adhered to, and once the tide of real war swept so near that the inhabitants of Domremy fled from their village in fear of their life, and remained in ambush for four days. Thus war was in the atmosphere she breathed, this sensitive, religious girl, with her mediæval belief in the near presence of saints and angels, her traditions of miracles, visions, and revelations. Not that she was a mere fanciful dreamer, but a strong-handed girl, used to field work, to following the plough, and wielding the spade. A short time before she set out on her mission she paid a fortnight's visit to a woman who kept an inn. There, it is said, she used to take the horses to the water, and learned to ride. Thus the physical qualifications she showed for the fatigues of the campaign cease to be surprising; she was by habit and training strong, active, and hardy. Some military genius, however, she must have possessed, for we are told that besides being an intrepid soldier she had the eye of a general, and was particularly skilful in the disposition of artillery—the weapon with which the peasant girl must have been least familiar.

Her visions commenced when she was thirteen years old. According to her deposition at her trial, the voices came always from the right side, and were accompanied by a light on the same side. She said also that she was fond of hearing the bells and that the voices of saints and angels mingled with the chimes. As the only reliable record of her career is given in Latin it is difficult to say what were the exact words which she believed she heard; but one of the angels' speeches has been thus retranslated: "Fille Dé, va, va, va; je serai à ton ayde; va" ("Daughter of God, go, go, go; I shall be your aid; go.") It is not difficult to see how such a reiterant phrase might sing itself to the melody of the bells in the ears of an imaginative girl to whom her country's troubles had been brought so close by personal experience. The first voices which Jeanne heard were doubtless the genuine impression of a sensitive and absorbed mind, but in the later and more elaborate visions one cannot but suspect the presence of self-delusion. It is difficult to say what was the first real impression that carried her away on her mission. According to her own statement, when she was about fifteen, she heard a boy's voice calling to her that her mother wanted her. She ran home, to find that she had not been sent for; but when she returned to rejoin her companions a bright cloud appeared before her eyes, and a voice said, "Jeanne, you must lead another life and do wonderful actions, for it is you whom the King of Heaven has chosen for the succour of France, and the help and protection of King Charles, expelled from his dominions. You will put on male attire, and taking arms, will be the leader of war. All things will be ruled by your counsel."

This is the statement which she made at her trial, years after the heavenly voice was heard, and when all things had taken place as she said they had been predicted. Without insinuating that La Pucelle was guilty of any taint of imposture, is it not possible that this was a case of prophesying after the event? How many of us "always foresaw" or "had an inward conviction" of something after circumstances have brought it about—convictions which were nebulous to a degree before circumstances did their work? Yet that Jeanne conceived herself to be inspired with a mission for the redemption of her country there can be no doubt, nor need we doubt the reality of the visions and the voice. It has been noted that these always came from the right side, and from this it has been inferred that Jeanne's was a case of unilateral insanity, that one side of the brain was faulty, while the other remained perfectly sane. Certainly a large proportion of sanity there must have been. The belief in her divine mission may have inspired herself to dauntless courage, and doubtless it impressed the common soldiers, and braced their courage to adventures which otherwise they would have deemed hopeless; but something more than the maid's belief in herself was needed to make the seasoned officers, Dunois, D'Alençon, and D'Anlon accept her advice against their own experience. They were not her blind followers, though they were undeniably impressed by the spectacle of a life which maintained its lofty ideals, its stainless purity amid all the rough life of a camp. They revered the woman who would have no swearing in her presence, and who, though she wore a man's armour and rode at the head of the troops, had a feminine hatred of the many dubious characters that followed in the train of the army. But they respected the shrewd leader that knew how to guide the conduct of a battle, and outwit the generalship of the enemy.

One would gladly accept the inspiration of Jeanne d'Arc as genuine, as her faith in it undeniably was; but it is evident that her voices deceived her sometimes. She predicted that she would set free the Duke of Orleans from his English captivity, and lead Charles VII. in triumph into Paris. She believed too that the heavenly powers with whom she was in communication would rescue her from her enemies. Certainly before the end of her brief career—a year in camps and a year in prison before, at the age of twenty, she was burned at Rouen—the over-confidence born of success was beginning to affect her judgment, as when she said that when she had driven the English out of France she would go to Bohemia to exterminate the Hussites, whom, as heretics, she disapproved of. Perhaps, had she lived longer, her hallucinations would have become more marked. For, looking at the matter from a modern point of view, it can hardly be doubted that the voices and the visions were hallucinations, however genuine was the patriotism that inspired them. It is not to every shepherd maiden that such visions come.

